# The Effective Force Constant Approach of Protein Flexibility Applied to Selected Photosynthetic Protein Complexes

**DOI:** 10.3390/molecules31020343

**Published:** 2026-01-19

**Authors:** Miriam Koppel, Maria Kulikova, Arina Sljusar, Mina Hajizadeh, Maksym Golub, Jörg Pieper

**Affiliations:** 1 Institute of Physics, University of Tartu, W. Ostwald Str. 1, 50411 Tartu, Estonia; 2 Institute Laue-Langevin Grenoble, 38042 Grenoble, France

**Keywords:** photosynthetic reaction center, light-harvesting complex, photoprotection, protein dynamics, effective force constant

## Abstract

Proteins are generally characterized by three-dimensional structures that are well suited for their specific function. It is much less accepted that a particular flexibility or plasticity of a protein is essential for performing its function. The latter plasticity encompasses the stochastic motions of small protein sidechains on the picosecond timescale that serve as “lubricating grease”, allowing slower functionally relevant conformational changes. Some remarkable examples of potential correlations between protein dynamics and function were observed for pigment–protein complexes in photosynthesis. For example, electron transfer and protein plasticity are concurrently suppressed in Photosystem II upon decreases in temperature or hydration, thus suggesting a prominent functional role of protein dynamics. An unusual dynamics–function correlation was observed for the major light-harvesting complex II, where the dynamics of charged protein residues affect the pigment absorption frequencies in photosynthetic light-harvesting. Generally, proteins exhibit a wide variety of motions on multiple time and length scales. However, there is an approach to characterize the plasticity of a protein as a single effective force constant that permits a straightforward comparison between different protein systems. Within this review, we determine the latter effective force constant for three photosynthetic proteins in different functional and organizational states. The force constant values determined appear to be rather different for each protein and are consistent with the requirements imposed by the various functions. These findings highlight the individual character of a protein’s flexibility and the role(s) it is playing for the specific function.

## 1. Introduction

**Figure 1:**
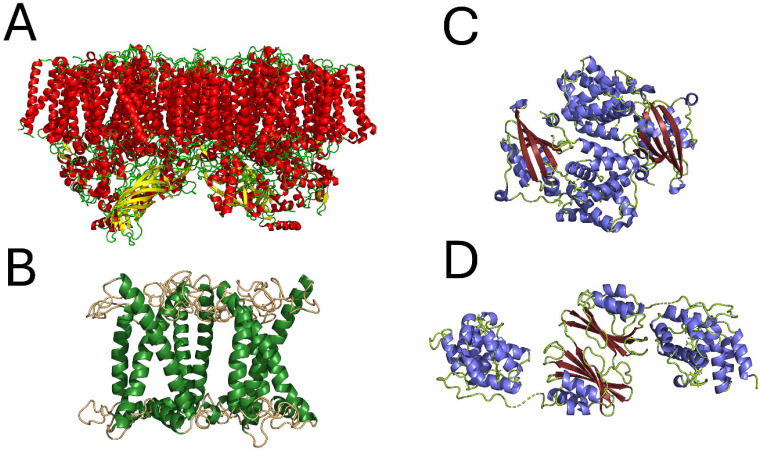
Crystal structures of the protein complexes considered in this review article: (Panel (**A**)): dimeric PSII (pdb 5KAF) according to Young et al. [1]; (Panel (**B**)): trimeric LHCII (pdb 1RWT) from Liu et al. [2]. The pigment molecules are omitted for clarity; (Panel (**C**)): structure of the dark-adapted state OCP^O^ (pdb 3MG1) from Wilson et al. [3]); (Panel (**D**)): structure of the active state OCP^R^ according to Dominguez-Martin et al. (pdb 7SCC) [4].

Photosystem II (PSII) (Figure 1A) [1], light-harvesting complex II (LHC II) (Figure 1B) [2], and the orange carotenoid protein (OCP) (Figure 1C,D) [3,4] are examples of photosynthetic pigment–protein complexes driving or regulating photosynthesis, i.e., the fundamental natural processes converting solar radiation into energy-rich sugars as the energy source for almost all life on earth. PSII is a multimeric membrane protein complex abundant in green plants and cyanobacteria that catalyzes light-induced water–water splitting releasing dioxygen [5], while LHCII is a membrane-intrinsic antenna protein complex of green plants, collecting solar radiation and transferring the resulting excitation energy to reaction center complexes [6]. In contrast, OCP is a water-soluble carotenoid-binding protein that provides photoprotection by interacting with the antenna system after being activated under intense light conditions in order to dissipate excess energy into heat [7]. Although the structures of the latter photosynthetic proteins are resolved at a nearly atomic resolution [1,2,3,4], a fully comprehensive picture of their function has not yet been achieved. One major reason for this shortcoming is the dynamical nature of some of the functional processes in these biomolecules, which require internal flexibility or plasticity of the respective proteins to allow larger-scale motions or conformational transitions essential for biological function.

Due to the hierarchical structure of biomolecules, their dynamics occur over a wide range of timescales, from femtoseconds to seconds, and over numerous length scales [8,9,10,11]. These dynamics include a variety of motions, ranging from the localized movements of small side chains (such as methyl group rotations) and adjustments of the protein backbone to fluctuations of entire domains and the global diffusion of whole macromolecules [12]. The stochastic, localized motions of small side chains enable the biomolecule to explore multiple subconformations on the picosecond timescale, which in turn promotes larger structural transitions that are critical for function [8,9]. The relationship between internal protein dynamics and function, often called the dynamics–function correlation, is a subject of significant scientific interest [13]. In photosynthetic proteins such as PSII, LHCII, and OCP, dynamics and plasticity are essential for their specific functions, enabling efficient energy transfer and charge separation in PSII [5], regulating exciton transfer and photoprotective quenching in LHCII [6], and controlling conformational activation in OCP [7]. While the precise relationship between internal dynamics and function depends on the protein, a decrease in molecular motions caused, for example, by lowering the temperature or hydration often coincides with decreased biological activity, highlighting the functional importance of protein plasticity [14,15,16,17].

Neutron spectroscopy is well-suited for studying protein dynamics because hydrogen atoms, which are abundant in biomolecules, have a very large incoherent neutron scattering cross-section (80.27 × 10^−28^ m^2^), and, therefore, neutrons are especially sensitive towards hydrogen [12,18]. Additionally, the energy of the neutrons used in scattering experiments is well-suited for studying various kinds of protein motions, while the accessible momentum transfers fall in the range of interatomic distances in biomolecules [18]. Depending on the spectral resolution, neutron scattering can probe protein motions across a wide range of timescales [12,18]. Hierarchical protein motions on nanosecond to microsecond timescales have also been observed using nuclear magnetic resonance spectroscopy [10], Mössbauer spectroscopy [19], and recent molecular dynamics simulations [20].

On the other hand, it is most understandable that the wide variety of protein motions abundant on multiple time and length scales leads to highly congested neutron spectra from bulk proteins, thus precluding a detailed (residue- or atom-resolved) analysis of protein motions [12,18]. In addition, neutron spectra reflecting multiple protein motions of a bulk protein within a given observation time window are analyzed by various theoretical approaches, ranging from the derivation of singular (“average”) relaxation times and the application of defined, yet phenomenological analytical models like rotation on or within a sphere to the determination of the distribution of relaxation processes (see Grimaldo et al. for a review [12]). In this regard, it is of high relevance that a method was proposed to express the plasticity of a whole protein in terms of a single numerical value of an effective force constant [21] that permits a straightforward comparison of the plasticity of proteins despite simplifying the inherent complexity of their dynamics. From elastic neutron scattering data, the average atomic mean square displacement (MSD) of proteins can be calculated, showing how much atoms fluctuate around their equilibrium positions. In the harmonic approximation, these thermal fluctuations can be interpreted with a simple spring model in which atoms move as if they were connected by effective harmonic springs. For a classical harmonic oscillator, the MSD increases linearly with temperature according to Equation (1) [21]:

(1) $$\langle u^{2}\rangle =\frac{k_{B}T}{\langle k\rangle }$$

where ⟨*u*^2^⟩ is the mean square displacement, *k*_B_ is the Boltzmann constant, *T* is the temperature, and ⟨*k*⟩ is the pseudo force or spring constant.

From the temperature dependence of MSD values, the resilience of a protein in a specific temperature range can be calculated following Equation (2) [21]:

(2) $$\langle k\rangle =\frac{2k_{B}}{d\langle u^{2}\rangle /dT}$$

where ⟨*k*⟩ is the pseudo force or spring constant, *k*_B_ is the Boltzmann constant, ⟨*u*^2^⟩ is the mean square displacement, and *T* is the temperature. In contrast to order parameters in nuclear magnetic resonance [22] and temperature B-factors in crystallography [23], the ⟨*k*⟩-value is an average parameter of the bulk protein and does not describe the flexibility of individual bonds or atoms, respectively.

The resulting pseudo force or spring constant ⟨*k*⟩ describes the effective plasticity of a protein even when protein motions are partially anharmonic. It is called a pseudo force constant because real biomolecular motions cannot be described by a single harmonic potential. Nevertheless, the constant still delivers a useful comparative measure of protein plasticity [21]. So far, this concept has not yet been applied to photosynthetic proteins, although the necessary neutron spectroscopy data are available for PSII, LHCII, and OCP [17,24,25,26,27]. In the present review, we briefly discuss the latter neutron spectroscopy results, determine the effective force constants ⟨*k*⟩ for different forms of PSII, LHCII, and OCP, and compare those ⟨*k*⟩-values to the literature data.

## 2. Protein Dynamics and Electron Transfer in PS II

Prominent examples for dynamics–function correlations are observed in PSII, a multimeric membrane protein occurring in the thylakoids of photosynthetic organisms [28]. PSII catalyzes the primary processes of photosynthesis. The high-resolution crystal structure of dimeric PSII [1] is shown in Figure 1A and appears to be valid in the solution state as well [29,30]. Light absorption by PSII triggers two fundamental reactions: water oxidation as a cyclic process involving five redox intermediates or S-states (S_0_–S_4_) and plastoquinone (PQ) reduction [31]. The latter light-driven redox reactions are largely dependent on temperature and hydration, which is often understood as an indication that conformational protein dynamics may play a key role in these functional processes in PSII [31].

**Figure 2:**
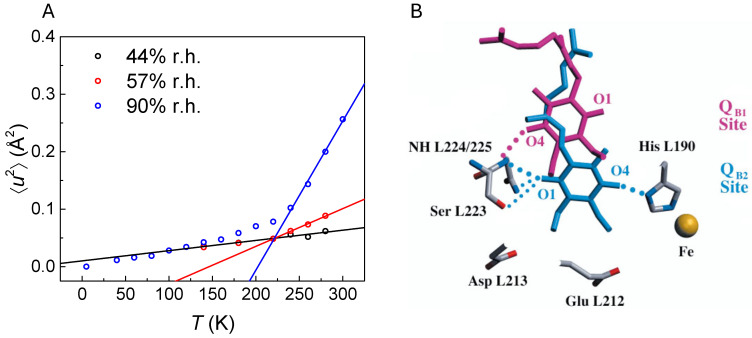
(**A**) Temperature dependence of MSD (⟨*u*^2^⟩) of PSII membrane fragments at 44% r.h. [17], 57% r.h. [17], and 90% r.h. [24] fitted using linear regression. The data were reproduced from Pieper et al. [17,24] with permission. (**B**) Conformational changes in the vicinity of the Q_B_ binding site upon Q_A_→Q_B_ electron transfer according to Stowell et al. [36]; see text. This figure is reprinted from Stowell et al. [36] with permission.

Correlations between protein dynamics and function are often assumed based on indirect observations, including the pronounced dependences of functional processes on, e.g., temperature and hydration. The idea behind such hypotheses is that an absence of conformational protein motions due to a temperature decrease or dehydration may hinder the occurrence of functionally relevant structural changes [9]. Focusing on the case of PSII, the light-induced primary charge separation is observed over a wide temperature range, including cryogenic temperatures [32], while both PQ reduction and specific S-state transitions exhibit pronounced temperature dependences. As an example, the electron transfer from the cofactor Q_A_ to the transiently bound acceptor Q_B_ becomes fully impaired below 200 K [19,33,34] and below a hydration level of about 44% relative humidity (r.h.) [35]. In the absence of direct measurements of protein dynamics, such effects were rationalized referring to the observation of a reorientation of the Q_B_ headgroup upon electron transfer based on X-ray crystallography, which would require a certain plasticity of the surrounding protein [36]. The inferred two conformations of the Q_B_ headgroup are depicted in violet and cyan colors in Figure 2B, respectively, along with the residues of the Q_B_ binding pocket. The rearrangements include the breaking and formation of hydrogen bonds. The idea of the latter relatively simple two-step gating mechanism, however, was later refined, inferring a more complex sequence of rearrangements of the protein domain surrounding the Q_B_ binding site depending on specific electron or proton uptake steps [37,38]. The critical role of protein dynamics for the binding of Q_B_ and its subsequent release after electron transfer was also inferred from molecular dynamics simulations [39]. Furthermore, the fine-tuning of protein dynamics through site-directed mutagenesis, i.e., the induction of more and more bulky protein residues affecting the rigidity of the protein matrix, was shown to lead to the thermal adaptation of electron transfer in thermophile bacteria [40]. As also mentioned above, the individual steps of water splitting by the water-oxidizing complex, referred to as S-state transitions, do also exhibit distinct dependences on temperature and hydration [41,42,43]. Therefore, understanding the correlation between protein dynamics and function is essential for a deeper comprehension of these processes.

Previously, neutron spectroscopy in the form of elastic and quasielastic neutron scattering (ENS and QENS, respectively) was employed to directly investigate the plasticity of hydrated PSII membrane fragments (PSIImf) [16,17,24]. The PSII plasticity was studied over a wide temperature range, starting from 5 K to eventually 300 K, revealing a dynamical transition at ~240 K for a relatively high hydration level of 90% r.h. [24]. The dynamical transition was reported to be strictly correlated with an increase in the temperature-dependent electron transfer efficiency from Q_A_ to Q_B_. At even higher temperatures, the plasticity of PSIImf revealed a further hydration-dependent transition between 310 K and 320 K, attributed to protein denaturation in the form of the disintegration of the oxygen-evolving complex [44]. At physiological temperatures, the plasticity of PSIImf becomes severely restricted below 44% r.h., along with the suppression of electron transfers in PSII upon dehydration [16]. Point mutations in close structural proximity of Q_A_ and Q_B_ also clearly influenced the overall plasticity of bacterial reaction centers, which display a high degree of structural similarity to PSII reaction centers [45,46]. QENS studies using PSIImf in solution revealed an even higher plasticity of PSIImf at physiological temperatures consistent with the higher hydration level [25].

We now turn to discuss the ⟨*u*^2^⟩-values of PSIImf shown in Figure 2A, which are reproduced from refs. [17,24] and which we subsequently use here to determine the related effective force constants ⟨*k*⟩ (Table 1). An inspection of Figure 2A shows that the plasticity of PSIImf hydrated at 57% r.h. differs in two distinct temperature ranges. While only relatively small ⟨*u*^2^⟩-values are observed below ~240 K, the ⟨*u*^2^⟩-values increase linearly with a much steeper slope above ~240 K. This can be interpreted as the dynamical transition, although observed at a much smaller extent than discussed above for a higher hydration level. This is in striking contrast to the ⟨*u*^2^⟩-values of PSIImf hydrated at 44% r.h., which increase linearly with rising temperatures within the entire temperature range considered. This means that no dynamical transition can be observed at a hydration level of 44% r.h., cf. the ⟨*u*^2^⟩-values of PSIImf hydrated at 57% and 90% r.h, respectively [24]. As discussed above, the latter transition is highly functionally relevant because it was found to be correlated with the temperature-dependent onset of electron transport from Q_A_ to Q_B_ [24].

The effective force constants ⟨*k*⟩ for PSIImf at four different hydration levels were determined from the ⟨*u*^2^⟩-values of PSIImf above the dynamical transition and are compiled in Table 1. An inspection of these values reveals that the force constants are gradually increasing with decreasing hydration levels, from 0.27 N/m in solution to a value as high as 15.5 N/m at 44% r.h., reflecting the increasing rigidity of PSIImf upon dehydration. The value in solution compares well with that of other ENS studies on membranes, e.g., purple membrane [47] and other experiments on PSIImf [44]. On the other hand, the resilience of purple membrane increases only from 0.05 N/m to 0.41 N/m upon complete dehydration, as we determined from the ⟨*u*^2^⟩-data of ref. [47]. This means that the resilience of PSIImf spans a remarkably larger range than that of purple membrane while remaining functionally fully intact. It has to be kept in mind, however, that the purple membrane stems from a halobacterium living in the Dead Sea, i.e., under rather stable hydration conditions, while PSIImf from green plants has to ensure proper functioning over a wide range of environmental conditions, including largely varying hydration conditions.

As discussed in refs. [17,24], the temperature dependence of electron transfer efficiency in PSIImf is well correlated with the onset of PSIImf plasticity when hydrated at 57% r.h. The latter effect further supports the observation of a hydration-dependent “dynamical transition” [24] and for a concomitant onset of PSIImf local conformational motions above 44% r.h. [16]. It has to be kept in mind, however, that the latter ENS data were measured using a different resolution sensitive to protein motions, at a time window of ~20 ps [17] compared with ~100 ps [24]. We conclude that a “freezing” of protein plasticity of PSIImf is observed upon dehydration, as well as upon temperature decrease, which is correlated with an inhibition of electron transfer between cofactors Q_A_ to Q_B_ in PSII. An analogous suppression of protein plasticity has been reported for a variety of proteins and protein–lipid systems [47,50,51,52]. It is interesting to note that the dynamics–function correlation observed for PSIImf is also apparent in bacterial reaction centers, although the transition temperature of the latter is lower [53]. It was inferred that the conformational changes underlying the electron transfer from Q_A_ to Q_B_ in bacterial reaction centers constitute an alteration of hydrogen bonds, including the reorientation of Ser223 in the vicinity of cofactors Q_A_ to Q_B_ [38,54]. The latter observations support the notion that protein motions on a picosecond timescale bear an important functional relevance for oxidative water splitting and PQ reduction in PSII, as well as in bacterial reaction centers initiated by a light-induced primary charge separation [55].

## 3. Protein Dynamics and Light-Harvesting in LHCII

Dynamics–function correlations are much less apparent in photosynthetic light-harvesting proteins because their function is dominated largely by electronic interactions between pigment molecules, while no large-scale structural changes are expected [56]. Light-harvesting proteins maintain their functional capabilities even at cryogenic temperatures [6,57,58]. The protein itself is often viewed as a simple “rigid scaffold” that exhibits entirely harmonic protein vibrations only, but no anharmonic conformational motions [57]. This is the reason why light-harvesting is comparatively well comprehended at non-physiological temperatures up to about 100 K [57,58]. As conformational dynamics become more abundant at higher temperatures, the spectroscopic properties of antenna proteins also exhibit unexpected changes above ~100 K [26,59]. These observations may point to an effect of protein dynamics on the functional properties of LHCII.

**Figure 3:**
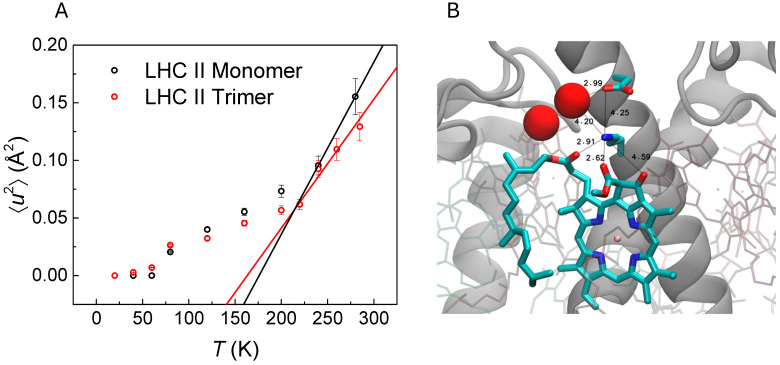
(**A**) Temperature dependence of MSD (⟨*u*^2^⟩) of LHCII monomer and trimer fitted using linear regression. The data were reproduced from Golub et al. [26] with permission. (**B**) Protein environment of Chl a 612 of LHCII. The charged residue Lys179 is able to form a hydrogen bond either with various Chl side chains or with close water molecules. Potential hydrogen bonds are illustrated by black lines and labeled by distances in Å. The figure is reproduced from Vrandecic et al. [59] with permission; Copyright American Chemical Society (ACS).

Conformational (localized) protein motions are reflected in temperature-dependent plots of the average atomic mean squared displacement ⟨*u*^2^⟩, as shown for monomeric and trimeric LHCII in Figure 3A (see Golub et al. [26]). Both datasets uncover dynamical transitions visible in the form of a drastic upturn of protein plasticity at about 240 K. We determined the effective force constants ⟨*k*⟩ of 1.85 N/m and 2.5 ± 0.3 N/m for monomeric and trimeric LHCII, respectively, from the data shown in Figure 3A. In Table 1, the values are compared to those of other proteins. Both ⟨*k*⟩-values of LHCII are higher than those of other proteins, listed for reference in Table 1. For example, bovine serum albumin (BSA), exhibiting a ⟨*k*⟩-value of only 0.55 N/m [48], is a soluble plasma protein with a high ligand binding capacity and acts as a transporter of various small molecules in serum [60]. It is reasonable to assume that the relatively high plasticity of BSA compared with LHCII is a prerequisite for efficient ligand binding. However, even the extremophile protein Hyperthermophilic malate dehydrogenase from *Methanococcus jannaschii* (*Mj* MalDH) found in archaea inhabiting deep sea hydrothermal vents [61,62] is characterized by a ⟨*k*⟩-value of 1.5 N/m, lower than for LHCII, which is linked with the high general resilience of *Mj* MalDH, necessary for adaptation to extreme temperatures [49]. That is, proteins adapted to different temperature regimes are expected to maintain a similar plasticity at their respective working temperatures following the “corresponding states” hypothesis [49]. It is remarkable that both LHCII forms considered here display a higher resilience than even a hyperthermophile protein like *Mj* MalDH. This must be related to the light-harvesting function of LHCII that does not require large structural changes, while proper distances between the pigment molecules bound by LHCII must be maintained for efficient excitation energy transfer.

According to the effective force constants, trimeric LHCII appears to be more rigid than the monomeric form. Consistent with the latter, the ⟨*u*^2^⟩-values of monomeric LHCII are larger than for trimeric LHCII above the dynamical transition at about 240 K. The apparent larger plasticity of monomeric LHCII is likely due to the larger solvent-exposed surface of the protein, resulting in a higher degree of mobility of the corresponding protein residues. In contrast, largely smaller ⟨*u*^2^⟩-values indicate a rather restricted plasticity of both LHCII forms due to the largely frozen conformational dynamics. As an exception, some flexibility is visible above ~80 K [59], which is independent of hydration or other solvent effects and is often attributed to the localized motions of methyl groups and similar small protein residues as an intrinsic property of the particular protein [63,64,65,66].

In summary, three different temperature ranges of protein dynamics are observed: (a) the protein is widely trapped in its conformational substates in the absence of internal dynamics below ~80 K; (b) the appearance of some localized protein motions above ~80 K; and (c) solvent-induced conformational protein motions on the picosecond timescale emerge above the dynamical transition at ~240 K in both forms of LHCII [18,67]. The latter transition temperature of ~240 K is in good agreement with that observed for fully hydrated PSIImf [24,68]. It is also consistent that NMR investigations of LHCII [69] and of entire thylakoid membranes [70] show vanishing NMR signals of the LHCII pigment molecules between 223 K and 244 K [69], thus indicating that the pigments became too flexible to be detected by NMR above the dynamical transition.

Despite the above considerations on protein plasticity, the functional role of LHCII flexibility in light-harvesting remains rather enigmatic. Vrandecic et al. [59] investigated this dynamics–function correlation using both neutron and optical spectroscopies, revealing that the positions of chlorophyll absorption bands appear to shift above similar transition temperatures reported above for protein dynamics [59]. Remarkably, the absorption bands appear stable below ~80 K but reveal a continuous red shift above. This effect cannot be understood based on the concept of a rigid protein structure, since all electrostatic pigment–pigment and pigment–protein interactions should persist in this case. Rather, an onset of picosecond dynamics above ~80 K encompassing the motions of charged residues close to pigment molecules may affect the transition frequencies of pigment molecules. The latter effect is illustrated in Figure 3B, showing the protein environment of chlorophyll a 612 of LHCII according to the crystal structure of Liu et al. [2]. Here, the charged residue Lys179 is able to form a hydrogen bond either with various chlorophyll side chains or with two close water molecules. Potential hydrogen bonds are illustrated by black lines and labeled with distances in Å in Figure 3B. Any thermally activated dynamical reorientation of the hydrogen bond formed by the latter charged residue varies the local electrostatic potential in the vicinity of the chlorophyll, thus affecting its transition energy due to pigment–protein interactions. This effect explains the observed temperature-dependent shifts in the LHCII transition energies and indicates a correlation between conformational dynamics and the position of excited electronic states of LHCII. It is also apparent that the structural fluctuations of protein sidechains occurring in LHCII are of a much smaller extent than the structural changes associated with electron transfer in PSII; see above.

## 4. Protein Dynamics in OCP

As pointed out above, the harvesting of solar energy is essential to initiate the primary processes of photosynthesis, which are performed by dedicated photosynthetic protein complexes [71]. However, the occurrence of high light intensities may lead to the damage of the photosynthetic apparatus or even to cell death due to the formation of reactive oxygen species (ROS) [72]. In order to counter such harmful conditions, cyanobacteria have developed a regulatory mechanism to prevent photodamage due to excessive light absorption, which is known as non-photochemical quenching (NPQ) [7,72,73,74,75]. Cyanobacterial NPQ relies on OCP to act as a high light sensor [76]. In the event of superfluous solar energy, OCP undergoes a significant structural transition towards its active state, binds to the phycobilisome (PBS) light-harvesting complexes, and eventually dissipates excess excitation energy into heat [77]. OCP is a water-soluble protein of about 35 kDa comprising a mixed α/β-fold C-terminal domain (CTD), as well as a second α-helical N-terminal domain (NTD) sharing one carotenoid pigment molecule in its central binding cavity (Figure 1C,D) [78]. In its dark-adapted state, OCP adopts a rather compact structure (Figure 1C), where both domains are closely bound by (a) the N-terminal extension (NTE) attached to the β-sheet region of the CTD, (b) a rather flexible linker interconnecting the two domains, (c) a salt bridge formed between the Arg155 and Glu244 residues, and (d) the forming hydrogen bonds between the carotenoid and the CTD residues Y201 and W288 [79]. OCP is capable of binding different carotenoid molecules including, among others, canthaxanthin (CAN) and echinenone (ECN). The OCP photocycle is initiated by blue light absorption transforming the orange, dark-adapted form OCP^O^ to the red, active form OCP^R^ (see Figure 1D), proceeding through a number of intermediate pigment–protein conformations [78]. Eventually, the active form of OCP is able to bind to the PBS light-harvesting complex, inducing the quenched conformation of PBS [4,78,80]. The latter PBS conformation including OCP^R^ is available at an almost atomic resolution [4]. However, the separation of the two OCP domains appears to be less pronounced when bound to PBS than reported before for the solution state, based on the low-resolution small angle scattering data of Golub et al. [81]. This means that an additional structural adaptation of OCP is necessary when binding to PBS, thus requiring an additional (dynamical) adaptation mechanism.

**Figure 4:**
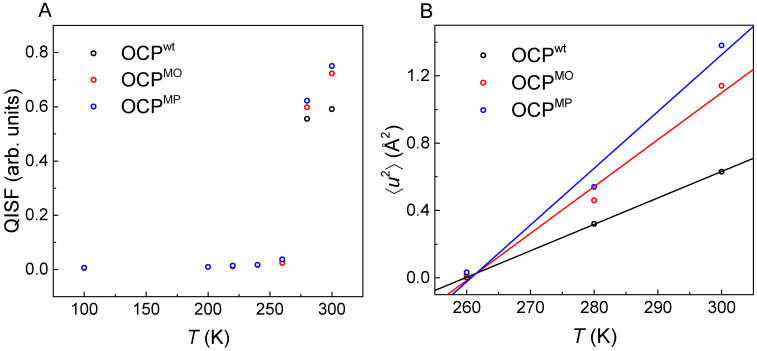
Temperature dependence of (**A**) quasielastic incoherent structure factor (QISF) of OCP^wt^, OCP^MO^, and OCP^MP^ and (**B**) MSD (⟨*u*^2^⟩) of OCP^wt^, OCP^MO^, and OCP^MP^ [27] fitted using linear regression. The data were reproduced from Hajizadeh et al., [27] Crystals 2024, 14 (4), 361. DOI: 10.3390/cryst14040361.

Besides well-adapted structural properties, the plasticity of proteins can be a decisive factor determining their functional capacities; see above [18]. This argument certainly applies to OCP, which exhibits significant conformational changes as a requirement for its function. However, the OCP active state also possesses a significantly enhanced protein flexibility, once coined as a “molten globule” state, in addition to its altered structure [82,83]. The enhanced protein plasticity of the active state was then directly observed using neutron spectroscopy at room temperature [84,85]. More recently, Hajizadeh et al. [27] employed neutron spectroscopy to study the dynamics of three different OCP forms over a wide temperature range between 100 K and 300 K, see Figure 4, which is the prerequisite to determine effective force constants. As a reference, OCP wild type (hereafter referred to as OCP^wt^) was investigated in the compact and dark-adapted forms. For comparison, two further OCP forms with point mutations at the residue W288 were used, differing in both structure and carotenoid content. OCP^W288A^-orange (hereafter referred to as OCP mutant orange or OCP^MO^) is anticipated to possess a compact structure, such as OCP^O^, and to contain ECN as a carotenoid, while OCP^288A^-pink (hereafter referred to as OCP mutant pink or OCP^MP^) resembles the elongated structure of the OCP active state, binding CAN as the carotenoid.

The temperature dependence of the QISF values of all three OCP samples determined by Hajizadeh et al. [27] are shown in Figure 4A. The QISF reveals thermally activated dynamics in the case of all three studied OCP forms above 200 K. This suggests that the protein preserves a certain plasticity even below solvent freezing, which is in line with the tightly bound hydration water surrounding the protein inducing residual protein mobility, even in the event of ice formation [25]. The significantly restricted plasticity of all three OCP forms below solvent freezing can be understood assuming that ice formation drives protein crowding, similar to aggregation, which may significantly restrict protein plasticity. In conclusion, we suggest that the low mobility of OCP in solutions below ~276 K is a result of ice-induced aggregation [25].

Because of the latter suppression of dynamics for OCP samples in solution, ⟨*u*^2^⟩-values were reported only in the temperature range 260–300 K; see Figure 4B. We have determined the effective force constants ⟨*k*⟩ for all three OCP forms and compiled them in Table 1 for comparisons with all other values. OCP exhibits generally much lower force constants than LHCII (see above), but also compared with the two model proteins BSA and *Mj* MalDH (see Table 1). This is consistent with the generally higher plasticity of OCP that is required to perform the structural adaptations that OCP undergoes when transitioning to the active state and when binding to PBS; see above.

In addition to the generally low resilience of all three OCP forms studied, it is remarkable that the effective force constants also differ significantly among the forms. The latter effects have to be discussed in terms of the expected structural differences between OCP^wt^ and the two mutants OCP^MO^ and OCP^MP^, respectively. In particular, the mutant OCP^MP^ is expected to have an elongated structure similar to the OCP active state. Thus, expecting a separation of CTD and NTDs as well as an unfolding of the NTE (see Figure 1D), OCP^MP^ is exhibiting a larger protein surface along with a concomitantly larger hydration, which permits a larger degree of mobility of the outer protein side chains. Furthermore, the unfolded NTE and flexible interdomain linker may contribute to an enhanced plasticity of this OCP form, resulting in the smallest effective force constant [84,85]. The latter difference between the dark-adapted and active states may be of high functional relevance because the plasticity of the OCP active state may be the decisive factor in adapting the OCP^R^ solution structure [81] to that observed in the quenched state of PBS [4].

However, it is also remarkable that the QISF of OCP^MO^ appears to be larger than that of OCP^wt^ but smaller than that of OCP^MP^ above solvent melting despite its expected compact structure. The larger plasticity of OCP^MO^ compared with OCP^wt^ can only be understood when bearing in mind that the mutation at residue W288A results in the absence of a hydrogen bond between the protein and the carotenoid, thus affecting the resilience of OCP in a wider region surrounding the site of mutation. Similar effects of point mutations were also observed in ref. [84]. On the other hand, the different plasticity of OCP^MP^ and OCP^MO^ may rather stem from different mutant structures. Another difference between the two mutant forms OCP^MP^ and OCP^MO^ lies in the number of carotenoid carbonyl groups, which determines the number of hydrogen bonds formed with the protein and with surrounding water molecules, thus affecting protein plasticity as discussed in detail by Hajizadeh et al. [27]. In summary, we conclude that the effective force constants determined for all three investigated OCP forms reflect their respective plasticity, which can be well understood in terms of their individual structures and the effect of the applied point mutations. We also conclude that, in the specific case of OCP, a significant structural change leads to a change in (functionally relevant) dynamics, while it is usually believed that picosecond dynamics serve as the “lubricating grease” allowing conformational changes.

## 5. Summary

In the present paper, we have applied the approach of an effective force constant [21] to characterize the plasticity of various forms of the three photosynthetic protein complexes PSII, LHCII, and OCP in terms of a single observable. Although this approach averages the plasticity of a whole protein, thus neglecting potential local differences, the effective force constant permits a straightforward comparison of the plasticities of individual proteins. The effective force constants ⟨*k*⟩ for PSIImf are gradually rising with decreasing hydration from 0.27 N/m in solution to the relatively high value of 15.5 N/m at 44% r.h., consistent with the strongly increasing resilience of PSIImf upon dehydration. It is remarkable that the resilience of purple membrane increases only from 0.05 N/m to 0.41 N/m upon complete dehydration. This means that the resilience of PSIImf spans a markedly larger range of effective force constants, while proper functioning (and thus allowing functionally relevant local structural changes, like in the vicinity of the electron acceptor Q_B_) is ensured over a wide range of hydration levels. The effective force constants ⟨*k*⟩ of 1.85 N/m and 2.5 ± 0.3 N/m derived for monomeric and trimeric LHCII, respectively, are also relatively high compared with other proteins. This high resilience of LHCII appears to be linked with the light-harvesting function of LHCII, which does not require large structural changes, while proper distances between the pigment molecules bound by LHCII must be maintained for efficient excitation energy transfer. In contrast, the effective force constants determined for three OCP forms are in the order of 0.08 N/m to 0.18 N/m and thus remarkably low, reflecting the high plasticity of OCP required to undergo the structural transitions necessary for its function. These examples underline that effective force constants are a relatively simple but effective means to characterize the flexibility of proteins. In summary, the three examples of photosynthetic proteins discussed here exhibit a remarkably different plasticity, reflected by their specific pseudo force constants: (i) PSII requires picosecond dynamics as a “lubricating grease” to allow a slower timescale and localized structural changes; (ii) LHCII is rather rigid because electronic interactions between bound pigments are most important and require a stable scaffold for light-harvesting; and (iii) OCP is highly flexible to permit the structural transition to the active state and adaptation to the phycobilisome binding site. These findings underline that the flexibility of proteins is a highly individual property of each protein that correlates well with their individual function.

## Figures and Tables

**Table 1:** Effective force constants ⟨*k*⟩ (N/m) for different photosynthetic protein complexes.

Sample	*T* (K)	⟨*k*⟩ (N/m)	Instrument	Resolution (μeV)	Reference
Effective force constants determined within this work *					
PSII membrane fragments 44% r.h.	220–280	15.5 ± 8.0	IN13	8	[17]
PSII membrane fragments 57% r.h.	220–280	4.20 ± 0.15	IN13	8	[17]
PSII membrane fragments 90% r.h.	240–300	1.07 ± 0.05	NEAT	93	[24]
PSII membrane fragments in solution	260–295	0.27 ± 0.02	IN6	88	[25]
LHCII monomer	220–280	∼1.85	NEAT	117	[26]
LHCII trimer	220–285	2.5 ± 0.30	NEAT	117	[26]
OCP^wt^	260–300	0.18 ± 0.01	Tof-Tof	75	[27]
OCP^MO^	260–300	0.10 ± 0.01	Tof-Tof	75	[27]
OCP^MP^	260–300	0.08 ± 0.01	Tof-Tof	75	[27]
Effective force constants from the literature					
PSII membrane fragments	280–310	0.12 ± 0.03 **	IN13	8	[44]
PSII membrane fragments	310–320	∼0.05 **	IN13	8	[44]
PSII membrane fragments	320–340	0.12 ± 0.05 **	IN13	8	[44]
Purple membrane dry	200–300	0.41 ± 0.04 ***	IN13	8	[47]
Purple membrane 75% r.h.	200–300	0.38 ± 0.03 ***	IN13	8	[47]
Purple membrane 86% r.h.	200–300	0.31 ± 0.02 ***	IN13	8	[47]
Purple membrane 93% r.h.	200–275	0.05 ± 0.01 ***	IN13	8	[47]
BSA in H_2_O	∼7–47	0.55 ± 0.246 **	IN13	8	[48]
*Mj* MalDH	∼7–47	1.5 **	IN13	8	[49]

* In this part of the table, the ⟨*k*⟩ values are calculated from the MSD values taken from referenced publications. ** These ⟨*k*⟩ values are taken from the referenced publications. *** These ⟨*k*⟩ values are calculated from the MSD values taken from referenced publications. BSA—bovine serum albumin. *Mj* MalDH—*M. jannaschii* malate dehydrogenase.

## Data Availability

No new data were created or analyzed in this study. Data sharing is not applicable to this article.
